# Computational design and evaluation of mRNA- and protein-based conjugate vaccines for influenza A and SARS-CoV-2 viruses

**DOI:** 10.1186/s43141-023-00574-x

**Published:** 2023-11-15

**Authors:** Amir Elalouf, Tomer Kedarya, Hadas Elalouf, Ariel Rosenfeld

**Affiliations:** 1 Department of Management, Bar-Ilan University, 5290002 Ramat Gan, Israel; 2 Information Science Department, Bar-Ilan University, 5290002 Ramat Gan, Israel

**Keywords:** Conjugate vaccine, Influenza A virus, SARS-CoV-2, Flurona, In silico vaccine

## Abstract

**Background:**

Israel confirmed the first case of “flurona”—a co-infection of seasonal flu (IAV) and SARS-CoV-2 in an unvaccinated pregnant woman. This twindemic has been confirmed in multiple countries and underscores the importance of managing respiratory viral illnesses.

**Results:**

The novel conjugate vaccine was designed by joining four hemagglutinin, three neuraminidase, and four S protein of B-cell epitopes, two hemagglutinin, three neuraminidase, and four S proteins of MHC-I epitopes, and three hemagglutinin, nine neuraminidase, and five S proteins of MHC-II epitopes with linkers and adjuvants. The constructed conjugate vaccine was found stable, non-toxic, non-allergic, and antigenic with 0.6466 scores. The vaccine contained 14.87% alpha helix, 29.85% extended strand, 9.64% beta-turn, and 45.64% random coil, which was modeled to a 3D structure with 94.7% residues in the most favored region of the Ramachandran plot and *Z*-score of −3.33. The molecular docking of the vaccine with TLR3 represented −1513.9 kcal/mol of binding energy with 39 hydrogen bonds and 514 non-bonded contacts, and 1.582925e-07 of eigenvalue complex. Immune stimulation prediction showed the conjugate vaccine could activate T and B lymphocytes to produce high levels of Th1 cytokines and antibodies.

**Conclusion:**

The in silico-designed vaccine against IAV and SARS-CoV-2 showed good population coverage and immune response with predicted T- and B-cell epitopes, favorable molecular docking, Ramachandran plot results, and good protein expression. It fulfilled safety criteria, indicating potential for preclinical studies and experimental clinical trials.

**Supplementary Information:**

The online version contains supplementary material available at 10.1186/s43141-023-00574-x.

## Background

SARS-CoV was initially detected in China in February 2003, triggering an outbreak that spread to four other countries. Conversely, MERS-CoV has been identified in dromedaries across several regions in the Middle East, Africa, and South Asia. Since 2012, 27 countries have reported cases of MERS-CoV, resulting in 858 documented deaths related to the infection and its complications. In addition, a novel coronavirus outbreak emerged in Wuhan, China, in December 2020 [1, 2]. Globally, more than 200 countries were affected by the first wave of the disease. The spread of COVID-19 was more apparent in the USA and Europe than in Africa [3]. SARS-CoV-2 was first detected in Israel on February 21st, 2020, and increased daily. A total of 1000 confirmed cases took about 30 days to emerge, followed by a 3-day doubling period [4]. As of July 29th, 2023, current coronavirus cases in Israel are 4,830,733, deaths are 12,585, and recovered patients are 4,798,473 [5, 6]. Globally, there have been a total of 692,196,348 confirmed cases of coronavirus infection. Among these cases, 664,486,884 individuals have successfully recovered, while 6,903,307 individuals, unfortunately, succumbed to the disease (as of July 29th, 2023) [6]. Similarly, influenza spreads and causes mild to severe symptoms. According to the WHO, 290,000–650,000 people die each year from this disease [7, 8]. A total of 2825 hospitalizations have been reported by the Israeli Center for Disease Control [9]. After the discovery of SARS-CoV-2 omicron in Israel, influenza A virus (IAV) cases sharply decreased in winter 2021–2022 compared to winter 2020. The SARS-CoV-2 alpha variant was more prevalent than influenza toward the end of 2020 [10]. The Israeli Outbreak Management Advisory Team coined the term “flurona” in late 2020. It is the first time Israel has confirmed cases of both seasonal flu and SARS-CoV-2 in an unvaccinated pregnant woman with mild symptoms [11]. These are similar infections referred to as the twin demic of two diseases [12]. Both are viral and cause breathing problems since they attack the upper respiratory tract. Coronavirus and influenza co-infections were reported in countries like the USA, Brazil, Hungary, the Philippines, and Israel [13, 14].

The primary symptoms of SARS-CoV-2 include severe cold and cough, shortness of breath, high fever, fatigue, loss of smell and taste, abdominal discomfort, loose motion, cognitive dysfunction, and other musculoskeletal, neurological, and cardiac problems that affect daily functioning [15]. The novel coronavirus can bind to angiotensin-converting enzyme 2 (ACE2) receptors, which are prominently expressed in the alveolar cells of the lungs, enterocytes, heart, liver, and kidney. This interaction can damage these organs and disrupt their normal functions, resulting in immunoinflammatory responses, such as cytokine storms [16, 17]. Mild to severe influenza symptoms include pulmonary symptoms, cough, and fever. It can cause serious complications, leading to death, particularly in young children, pregnant women, and the elderly [18]. A study suggests that infections caused by the wild-type SARS-CoV-2 virus may lead to higher mortality rates compared to respiratory viruses commonly prevalent during winter [19]. SARS-CoV-2, influenza, and other respiratory viruses can result in severe complications, including cardiovascular events and bacterial co-infections that may lead to death [20]. However, the immune system can simultaneously produce antibodies for multiple pathogens so that a dual infection could trigger an even more robust defense response [10].

During the initial stages of the COVID-19 pandemic, existing antivirals, antibacterials, antimalarials, mucolytic agents, and antipyretic paracetamol were used to manage infected patients. However, specific small-molecule chemotherapeutics were limited, with only a few emergency-use vaccines available. Consequently, researchers explored the repurposing of US Food and Drug Administration (FDA)-approved chemotherapeutics to combat the virus [21]. In a study, 34 drugs, including antivirals and antimalarials, were subjected to in silico molecular docking analyses to assess their potential affinity for inhibiting the COVID-19 protease target. Thirteen compounds exhibited promising binding affinities against the protease, suggesting their potential as anti-COVID agents [22]. Additionally, a regression model validated the predicted activities of various compounds. High-throughput screening identified bedaquiline, lefamulin, dexamethasone, and cefixime as promising candidates for repurposing against COVID-19. Meanwhile, doxycycline, cefpodoxime, ciprofloxacin, sparfloxacin, moxifloxacin, and TBAJ-876 displayed moderate binding affinities. These findings underscore the potential of repurposing chemotherapeutics as effective treatments for COVID-19 and provide valuable insights for further clinical investigations [23].

Vaccines play a crucial role in controlling the SARS-CoV-2 pandemic [24]. The vaccination drive against SARS-CoV-2 was initiated in December 2020 with the Pfizer/BioNTech BNT162b2 mRNA vaccine, approved by the WHO [15]. FDA authorized the Pfizer/BioNTech vaccine in August 2021 as the first vaccine to prevent SARS-CoV-2 infection in adults [25]. By October 2021, the WHO had authorized the use of vaccines by Pfizer/BioNTech, Serum Institute of India, Sinopharm, Janssen, AstraZeneca-SK Bio, and Moderna for emergency use [26]. The vaccines presently approved by the FDA comprise the Pfizer-BioNTech and Moderna COVID-19 vaccines, classified as mRNA vaccines, and the Novavax COVID-19 vaccine, categorized as a protein subunit vaccine [27]. As of January 2022, approximately 58% of the global population had received SARS-CoV-2 vaccines, with 9.2 billion doses administered [28]. Evidence shows that these vaccines can prevent severe complications and death, even against more contagious variants like Delta and Omicron SARS-CoV-2 [29]. COVID-19 vaccines induce a form of host active immunity that may not confer long-term protection uniformly across all individuals. The duration of this immunity is subject to variation and relies on diverse factors, including an individual’s age, health condition, and the specific type of vaccine administered [30]. During 2020–2022, COVID-19 vaccine drawbacks included limited long-term safety data, reports of adverse reactions (e.g., allergic responses, blood clotting disorders), and reduced efficacy against specific variants, necessitating vaccine adaptation [31, 32]. Computational tools have facilitated the development of a novel multi-epitope vaccine against 2019-nCoV, which can induce CD4+ and CD8+ T-cell responses to achieve a comprehensive and effective immune response [33]. CD4+ T-cells are vital in coordinating and activating other immune cells, including B-cells that produce antibodies. CD8+ T-cells, on the other hand, directly target and eliminate virus-infected cells [34]. In silico assessment demonstrated that the designated chimeric protein could simultaneously stimulate humoral and cellular immunity [33].

As viruses evolve rapidly, seasonal influenza vaccines must be updated periodically to remain effective and save lives yearly. For centuries, influenza has contributed significantly to mortality and threatened public health. An influenza vaccine stimulates an antibody response against circulating viruses. The trivalent vaccine includes influenza strains A/H1N1 and A/H3N2 and the predominant B strain [35]. The recombinant multi-epitope vaccine was perceived to have unique and suitable immunologic properties. In silico immunogenicity tests can be performed against the influenza virus using this recombinant multi-epitope vaccine expressed in the prokaryotic system [36]. Another in silico analysis was performed to identify the IAV subtypes with the most antigenically significant T- and B-cell epitopes from N1 to N4 and N6 to N8. Recombinant vaccines, cell culture-grown viruses, and adjuvanted vaccines have also been licensed in recent years [37].

The mRNA technology has shown remarkable efficacy worldwide during the SARS-CoV-2 outbreak and is now positioned to provide a new series of high-performing vaccines [38]. It may be easier to match strains because eggs will not have to be grown. Using mRNA can also improve pharmaceutical manufacturing since mRNA production is less complex than recombinant technology [39]. Because human cells accurately produce viral proteins, vaccine efficacy may be improved or expanded. Moreover, mRNA allows for the inclusion of more antigens, potentially boosting cellular immunity. The mRNA flu vaccine has been in progress for 10 years, and both H10N8 and H7N9 vaccines have demonstrated proof-of-concept in healthy adults [40].

Vaccines using the conjugate method combine weak antigens with strong antigens so the immune system can react sturdier [41]. Several examples include the *Haemophilus influenzae* type b and typhoid conjugate vaccines. It is possible to achieve marked herd immunity with conjugate vaccines due to their ability to elicit immunogenic memory and reduce the asymptomatic transmission of the bacteria [42]. SARS-CoV-2 receptor binding domains are site-selectively functionalized and chemically conjugated to highly immunogenic carrier proteins to produce conjugates that have immunological activity [43]. SOBERANA 02 is a recombinant receptor binding domain conjugated to the tetanus toxoid vaccine, involved in T-cell response and neutralizing the IgG [44].

The objective of this study was to create a conjugate vaccine that is safe and hypoallergenic using artificial intelligence. The vaccine was constructed of several epitopes from the neuraminidase (NA) and hemagglutinin (HA) proteins of IAV, as well as spike (S) proteins from SARS-CoV-2. The ensuing vaccine was analyzed using bioinformatic tools to assess its interaction with receptors activating the immune system and to examine its immunogenic properties in real-world scenarios.

## Methods

### Protein sequence retrieval and multiple sequence alignment

The sequences of NA and HA of IAV and S protein of SARS-CoV-2 were downloaded from UniProt (https://www.uniprot.org/) and NCBI (https://www.ncbi.nlm.nih.gov/) by applying a search filter specific for Israel. Constraint-based Multiple Alignment Tool (COBALT) (https://www.ncbi.nlm.nih.gov/tools/cobalt/re_cobalt.cgi) was employed to estimate the conserved regions of all the downloaded sequences of the respective proteins by multiple sequence alignment with local sequence similarity and conserved domain information.

### Antigenic proteins

VaxiJen v2.0 server (http://www.ddg-pharmfac.net/vaxijen/VaxiJen/VaxiJen.html) [45–47] estimated the antigenicity of multiple sequence alignment of NA and HA of IAV and S protein of SARS-CoV-2.

### Transmembrane topology prediction

TMHMM 2.0 (Transmembrane helices hidden Markov models) (https://services.healthtech.dtu.dk/services/TMHMM-2.0/) [48, 49] was utilized for the identification of the inner, outer, and transmembrane helical region of multiply aligned sequences of NA and HA of IAV and S protein of SARS-CoV-2.

### Physiochemical properties

ExPASy-ProtParam online web server (https://web.expasy.org/protparam/) [50, 51] was utilized to evaluate the physiochemical properties of all the selected sequences of the NA and HA of IAV and S protein of SARS-CoV-2. ProtParam computes different physical and chemical parameters for protein sequences or stored proteins in Swiss-Prot or TrEMBL. The parameters computed include the number of amino acids, theoretical isoelectric point (pI), aliphatic index (AI), molecular weight (Da), extinction coefficients (Ec) (M-1 cm-1, at 280nm), grand average of hydropathicity (GRAVY), instability index (II), estimated half-life, negatively charged residues (Asp + Glu) (R-), and positively charged residues (Arg + Lys) (R+).

### Epitopes prediction and important features profiling

Immune Epitope Database (IEDB) (https://www.iedb.org/) [52] bioinformatics database tool was exploited for the prediction of T- and B-cells epitopes. IEDB used different prediction methods, such as Ab initio prediction, homology-based prediction, B-cell and T-cell epitope prediction, and structure-based prediction, to predict, curate, and validate the epitopes with data through literature and experiments. In this study, MHC-I binding prediction server (http://tools.iedb.org/mhci/) predicted the conserved MHC-I binding epitopes using the NetMHCpan EL 4.1 method [53] of the sequences of NA and HA of IAV and S protein of SARS-CoV-2. In addition, the FASTA sequences of NA and HA of IAV and S protein of SARS-CoV-2 were submitted separately in the MHC-I binding prediction server by setting the human as the MHC source species and 9-mer HLA allele reference set.

Similarly, MHC-II binding prediction server (http://tools.iedb.org/mhcii/) predicted the conserved binding epitopes of MHC-II using the IEDB recommended 2.22 method [52] of the sequences of NA and HA of IAV and S protein of SARS-CoV-2. The FASTA sequences of NA and HA of IAV and S protein of SARS-CoV-2 were submitted separately in the MHC-II binding prediction server by setting the human as source species, HLA-DR, HLA-DQ, and HLA-DP as locus, full HLA reference set, and default 15-mer epitopes length.

The antibody epitope prediction web-server (http://tools.iedb.org/bcell/) predicted the conserved epitopes of B-cells using the BepiPred Linear Epitope Prediction 2.0 method [54] of the sequences of NA and HA of IAV and S protein of SARS-CoV-2, separately.

The results obtained from these servers were arranged in an excel spreadsheet. Each predicted epitope of all the sequences was evaluated to be part of the conserved sequence and present outside the transmembrane of the viruses. Then, each epitope was feature profiled to predict their antigenicity, toxicity, and allergenicity by using online servers such as VaxiJen v2.0 [45–47], ToxinPred2 (https://webs.iiitd.edu.in/raghava/toxinpred2/batch.html) [55], and AllerTOP v2.0 (https://www.ddg-pharmfac.net/AllerTOP/) [56] servers, respectively.

The Population Coverage calculation is a tool that estimates the percentage of a population predicted to be covered by a given epitope or set of epitopes. The calculation assumes that specific epitopes are more conserved across different individuals than others and, thus, are more likely to be recognized by the immune system in a larger population. The tool uses information from the IEDB’s database of experimentally determined epitopes and data from population genetic studies to predict the population coverage of a given epitope or set of epitopes. IEDB’s Population Coverage (http://tools.iedb.org/population/) [57] was used to calculate the population coverage of individual epitopes of MHC class-I and class-II by selecting the Israeli population.

### Epitope conservancy analysis

The selected antigenic epitopes of the sequences of NA and HA of IAV and S protein of SARS-CoV-2 were analyzed for the conservancy by epitope conservancy analysis [58].

### Vaccine construction

The antigenic epitopes of MHC-I, MHC-II, B-cells, and adjuvant were linked with AAY, GPGPG, KK, and EAAAK linkers [59–61], respectively, to construct a combined vaccine for both IAV and SARS-CoV-2 viruses. The sequence of the constructed vaccine started with a 50S ribosomal adjuvant protein (UniProtKB: P60438) [62, 63] following the linkers and epitopes of sequences of NA and HA of IAV and S protein of SARS-CoV-2 and ended with MITD (MHC I-targeting domain) sequence (UniProt ID: Q8WV92) [62–64] and 6-His tag [65].

### Physiochemical parameters, antigenicity, allergenicity, toxicity, and solubility of vaccine construct

ExPASy-ProtParam [50, 51] online web server was used to analyze the chemical and physical parameters of constructed conjugate vaccine. The antigenicity, allergenicity, and toxicity of the constructed vaccine were evaluated using online servers VaxiJen 2.0 [45–47], AllerTop 2.0 [56], and Toxinpred2 [55], respectively. SoluProt [66] predicted the soluble protein expression in *Escherichia coli*. Solubility scores higher than 0.5 indicate that the expression is soluble, whereas scores lower than 0.5 indicate that the expression is insoluble.

### Secondary and tertiary structures modeling

Various variables of secondary structures of the combined vaccine construct of IAV and SARS-CoV-2 viruses were anticipated using an online sever SOPMA (https://npsa-pbil.ibcp.fr/NPSA/npsa_sopma.html) [67]. All the SOPMA features were fixed to default values, which included the use of 4 conformational states such as helix, sheet, turn, and coil of secondary structure, a window width of 17, and a similarity threshold of 8. For detailed information on vaccine secondary structure, graphically, PSIPRED (PSI-blast-based secondary structure PREDiction) (http://bioinf.cs.ucl.ac.uk/psipred/) [68–70] was utilized to get the secondary structure of conjugate vaccine construct of IAV and SARS-CoV-2 viruses. Furthermore, the tertiary structure of the conjugate vaccine construct was then anticipated by ColabFold (https://colab.research.google.com/github/sokrypton/ColabFold/blob/main/AlphaFold2.ipynb#scrollTo=iccGdbe_Pmt9) [71], which uses AlphaFold2 and Alphafold2-multimer by generating sequence templates through HHsearch and MMseqs2.

### Refining and verifying the 3D structure of vaccine

The predicted 3D structure of the vaccine construct was refined using GalaxyRefine (https://galaxy.seoklab.org/cgi-bin/submit.cgi?type=REFINE) [72] online server. This server repacks and rebuilds the side chains by molecular dynamics simulation (MDS) procedure to relax the structure. Furthermore, the 3D structure of the vaccine construct was verified by PROCHECK (https://saves.mbi.ucla.edu/) [73], which assesses the stereochemical integrity of the vaccine structure by evaluating overall structure geometry and residue-by-residue geometry to construct the Ramachandran Plot. In addition, a *Z*-score was computed for each modeled vaccine structure using the ProSA tool (https://prosa.services.came.sbg.ac.at/prosa.php) [74]. By utilizing the *Z*-score, a comparison was made between the 3D modeled structure of the vaccine and protein structures obtained through experimental methods such as NMR (nuclear magnetic resonance) and X-ray crystallography. The degree of similarity of the modeled structure with native proteins of similar size was evaluated using QMEAN *Z*-scores (https://swissmodel.expasy.org/qmean/) based on experimental structures. This estimation includes interatomic packing, backbone geometry, and unexpected solvent accessibility [74, 75].

### Discontinuous B-cell epitope prediction

ElliPro tool (http://tools.iedb.org/ellipro/) [76] of the IEDB server confirmed the presence of linear and conformational B-cell epitopes in the constructed conjugated vaccine.

### Molecular docking and molecular simulation

To inspect the binding affinity of the vaccine with the Toll-like receptor 3 (TLR3), protein-protein molecular docking was performed by using ClusPro 2.0 (https://cluspro.bu.edu/login.php) [77–80]. The 3D structure of TLR3 was downloaded from Protein Data Bank (PDB) using PDB ID: 2A0Z. Discovery Studio removed all the ligands and other heteroatoms from the TLR3 structure. Then, the cleaned TLR3 protein as a receptor and vaccine as a ligand were uploaded to the ClusPro 2.0 server for protein-protein docking [81, 82]. The best vaccine-TLR3 docked result was further used for the MDS using an online server iMODS (https://imods.iqfr.csic.es/) [83, 84]. iMODS server analyzes the NMA (normal mode analyses) to determine collective motion in internal coordinates and torsional angles of the vaccine-TLR3 complex. Other than costly atomistic simulations, essential dynamics were used to stabilize proteins and predict immanent motions of the complexes and their magnitudes based on B-factors, covariance, deformability, and eigenvalues.

### Codon optimization and in silico cloning

For the efficient expression analysis of the vaccine in *E. coli* K12, JCAT (Java Codon Adaptation Tool) (http://www.jcat.de/) [85] was utilized for the codon adaptation to verify the binding site of prokaryotic ribosomes, identify rho-independent transcription termination sites, and ascertain the locations of restriction enzyme cleavage sites. Then, SnapGene 4.2 software [82, 86] was used for the cloning process. SgrAI and HpaI restriction sites (present in both vector and vaccine) were instigated in the vaccine sequence to the C- and N-terminal, respectively. The constructed vaccine was ultimately imbedded into the *E. coli* pET28a(+) expression vector for the cloning simulation.

### mRNA vaccine secondary structure prediction

For the anticipation of the secondary structure of mRNA of the vaccine, firstly Transcription and Translation Tool (https://biomodel.uah.es/en/lab/cybertory/analysis/trans.htm) [81] was used to convert the optimized DNA sequence to RNA sequence. Then, a web server RNAfold (http://rna.tbi.univie.ac.at/cgi-bin/RNAWebSuite/RNAfold.cgi) [87–89] was utilized for the mRNA secondary structure prediction thermodynamically and with minimal free energy score.

### Immune simulation analysis

For the realistic immunogenic profile of the constructed conjugate vaccine, an antigen-based immune simulator online web server C-ImmSim (https://kraken.iac.rm.cnr.it/C-IMMSIM/) [90, 91] was used to predict the immune reactions with the combination of the machine learning and position-specific scoring matrix (PSSM) algorithm. Most vaccines require a 4-week gap between two doses as a standard rule. A total of three injections of 1000 antigens were injected at 8-week and 24-week intervals after the initial injection, at 168- and 504-time points, respectively. Each time point represents an 8-h interval in reality, and the first time point corresponds to the injection at time zero. The simulation involved 1050 steps, with the other parameters remaining at default. The generated figures were interpreted as the Simpson’s Diversity Index (D) [81, 92].

## Results

### Protein sequence retrieval and multiple sequence alignment

The sequences of NA and HA of the IAV and S protein of SARS-CoV-2 were chosen after multiple sequence alignments to identify the conversed sequences for the vaccine development.

### Antigenic proteins

VaxiJen v2.0 validated the antigenicity of the NA and HA of IAV and S protein of SARS-CoV-2 sequences. The sequences of NA and HA of the IAV showed 0.527 and 0.5039 antigenicity scores at the threshold level of 0.4, respectively. Similarly, the S protein sequence showed an antigenicity score of 0.4646 at the threshold level of 0.4.

### Transmembrane topology prediction

For the vaccine candidate, selected epitopes of the proteins for the immune response must be exposed. Hence, transmembrane topology analysis of NA and HA of IAV and S protein of SARS-CoV-2 by TMHMM 2.0 web server revealed the exo-, trans, and endo-membrane amino acids length, shown in Table 1.

**Table 1:** Transmembrane topology of the NA and HA of IAV and S protein of SARS-CoV-2

Protein	Virus	Position	Amino acid
**HA**	IAV	Outside	1–527
		TM helix	528–550
		Inside	551–563
**NA**	IAV	Outside	30–429
		TM helix	7–29
		Inside	1–6
**S protein**	SARS-CoV-2	Outside	1–1213
		TM helix	1214–1236
		Inside	1237–1273

### Physiochemical properties

ProtParam calculated the physiochemical properties of the selected sequences of NA and HA of IAV and S protein of SARS-CoV-2 (as shown in Table 2).

**Table 2:** Physiochemical properties of the selected sequences of NA and HA of IAV and S protein of SARS-CoV-2 predicted by ProtParam

Sr. No.	Physiochemical properties	HA	NA	S protein
**1**	Number of amino acids	563	429	1273
**2**	Mol. wt. (Da)	63,644.01	46,810.07	141,178.47
**3**	Theoretical pI	6.05	5.48	6.24
**4**	Ec (M^−1^ cm^−1^, at 280nm)	91,635	10,0015	148,960
**5**	GRAVY	−0.418	−0.098	−0.079
**6**	II	35.63	29.09	33.01
**7**	AI	82.08	78.39	84.67
**8**	R^+^	58	32	103
**9**	R^-^	66	39	110
**10**	Estimated half-life	30 hours (mammalian reticulocytes, in vitro), >20 hours (yeast, in vivo), and >10 hours (*E. coli*, in vivo).	30 hours (mammalian reticulocytes, in vitro), >20 hours (yeast, in vivo), and >10 hours (*E. coli*, in vivo).	30 hours (mammalian reticulocytes, in vitro), >20 hours (yeast, in vivo), and >10 hours (*E. coli*, in vivo).

### Epitopes prediction and important features profiling

An IDEB server was employed to predict MHC-I, MHC-II, and B-cells binding epitopes of NA and HA of IAV and S protein of SARS-CoV-2. With the NetMHCpan EL 4.1 method, the IDEB server predicted 14,013, 11,232, and 32,535 MHC-I binding epitopes of the sequences of NA and HA of IAV and S protein SARS-CoV-2 that were available outside the transmembrane of the viruses, respectively. In parallel, IEDB recommended 2.22 method predicted 9763, 11,070, and 32,372 MHC-II binding epitopes of the sequences of NA and HA of IAV and S protein of SARS-CoV-2 that were also present outside the transmembrane of the viruses, respectively. Lastly, the BepiPred Linear Epitope Prediction 2.0 method also predicted the B-cell epitopes of NA and HA of IAV and S protein of SARS-CoV-2. All the MHC-I, MHC-II, and B-cell binding epitopes of NA and HA of IAV and S protein of SARS-CoV-2 were sorted with their IC50 values and then filtered with having conserved antigenic, non-allergic, and non-toxic sequences, shown in Tables 3, 4 and 5, respectively. The alleles of all the selected epitopes of MHC-I and MHC-II are shown in Tables S1 and S2 from supplementary material. Additionally, the percentages of Israeli population coverage of MHC-I and MHC-II epitopes were determined using IEDB’s Population Coverage, as in Tables 3 and 4.

**Table 3:** Antigenic MHC-I binding epitopes of NA and HA of IAV and S protein of SARS-CoV-2 predicted by NetMHCpan EL 4.1 method using IEDB server

Sr. No.	Proteins	Position	Peptides	Antigenicity score	Toxicity	Allergenicity	Population coverage (%)
**1**	HA	490–498	MESVRNGTY	0.8219	No	No	89.39
		448–456	TLDFHDSNV	1.4584	No	No	89.39
**2**	NA	132–140	TVKDRSPYR	0.9523	No	No	89.39
		150–158	APSPYNSRF	0.7486	No	No	89.39
		329–337	GVKGFSYRY	0.6322	No	No	89.39
**3**	S protein	714–722	IPTNFTISV	0.882	No	No	89.39
		718–726	FTISVTTEI	0.8535	No	No	89.39
		89–97	GVYFASTEK	0.7112	No	No	89.39
		680–688	SPRRARSVA	0.7729	No	No	89.39

**Table 4:** Antigenic MHC-II binding epitopes of NA and HA of IAV and S protein of SARS-CoV-2 predicted by IEDB recommended 2.22 method using IEDB server

Sr. No.	Proteins	Position	Peptides	Antigenicity score	Toxicity	Allergenicity	Population coverage (%)
**1**	HA	269–283	INSSMPFHNIHPLTI	**0.9172**	No	No	68.79
		270–284	NSSMPFHNIHPLTIG	**1.397**	No	No	68.79
		403–417	TYNAELLVLMENERT	**0.6447**	No	No	68.79
**2**	NA	115–129	TFFLTQGALLNDKHS	**0.6999**	No	No	68.79
		330–344	VKGFSYRYGNGVWIG	**0.4729**	No	No	68.79
		329–343	GVKGFSYRYGNGVWI	**0.5514**	No	No	68.79
		368–382	DSRFSVRQDVVAMTD	**0.6503**	No	No	68.79
		370–384	RFSVRQDVVAMTDRS	**0.6606**	No	No	68.79
		369–383	SRFSVRQDVVAMTDR	**0.5961**	No	No	68.79
		367–381	TDSRFSVRQDVVAMT	**0.6988**	No	No	68.79
		332–346	GFSYRYGNGVWIGRT	**1.3029**	No	No	68.79
		333–347	FSYRYGNGVWIGRTK	**1.382**	No	No	68.79
**3**	S protein	115–129	QSLLIVNNATNVVIK	**0.4343**	No	No	68.79
		510–524	VVVLSFELLHAPATV	**0.8083**	No	No	68.79
		509–523	RVVVLSFELLHAPAT	**0.7485**	No	No	68.79
		508–522	YRVVVLSFELLHAPA	**0.7072**	No	No	68.79
		233–247	INITRFQTLLALHRS	**0.4118**	No	No	68.79

**Table 5:** Antigenic B-cell epitopes of NA and HA of IAV and S protein of SARS-CoV-2 anticipated by BepiPred Linear Epitope Prediction 2.0 method using IEDB server

Sr. No.	Proteins	Position	Peptides	Length	Antigenicity score	Toxicity	Allergenicity
**1**	HA	490–514	MESVRNGTYDYPQYSEEARLKREEI	25	**0.5149**	No	No
		97–155	EKISPANDLCYPGNFNDYEELKHLLSRINHFEKIHIIPKESWSYHEASGVSSACPYQGR	59	**0.5389**	No	No
		167–183	KKNDAYPTIKKSYNNTN	17	**0.8349**	No	No
		329–341	ATGLRNSPQGEKR	13	**0.9706**	No	No
**2**	NA	39–51	QTGNQNHTGICNQ	13	**0.9098**	No	No
		60–77	AGQDSTSVILTGNSSLCP	18	**0.5546**	No	No
		435–449	DTVDWSWPDGAELPF	15	**0.9144**	No	No
**3**	S protein	138–156	DPFLGVYYHKNNKSWMESE	19	**0.5959**	No	No
		206–221	KHTPINLVRDLPQGFS	16	**0.6403**	No	No
		313–322	YQTSNFRVQP	10	**1.1866**	No	No
		369–393	YNSASFSTFKCYGVSPTKLNDLCFT	25	**1.4031**	No	No

### Epitope conservancy analysis

Epitope conservancy analysis confirmed the conservancy of all the selected epitopes for constructing a vaccine of NA and HA of IAV and S protein of SARS-CoV-2.

### Vaccine construct

**Fig. 1:**
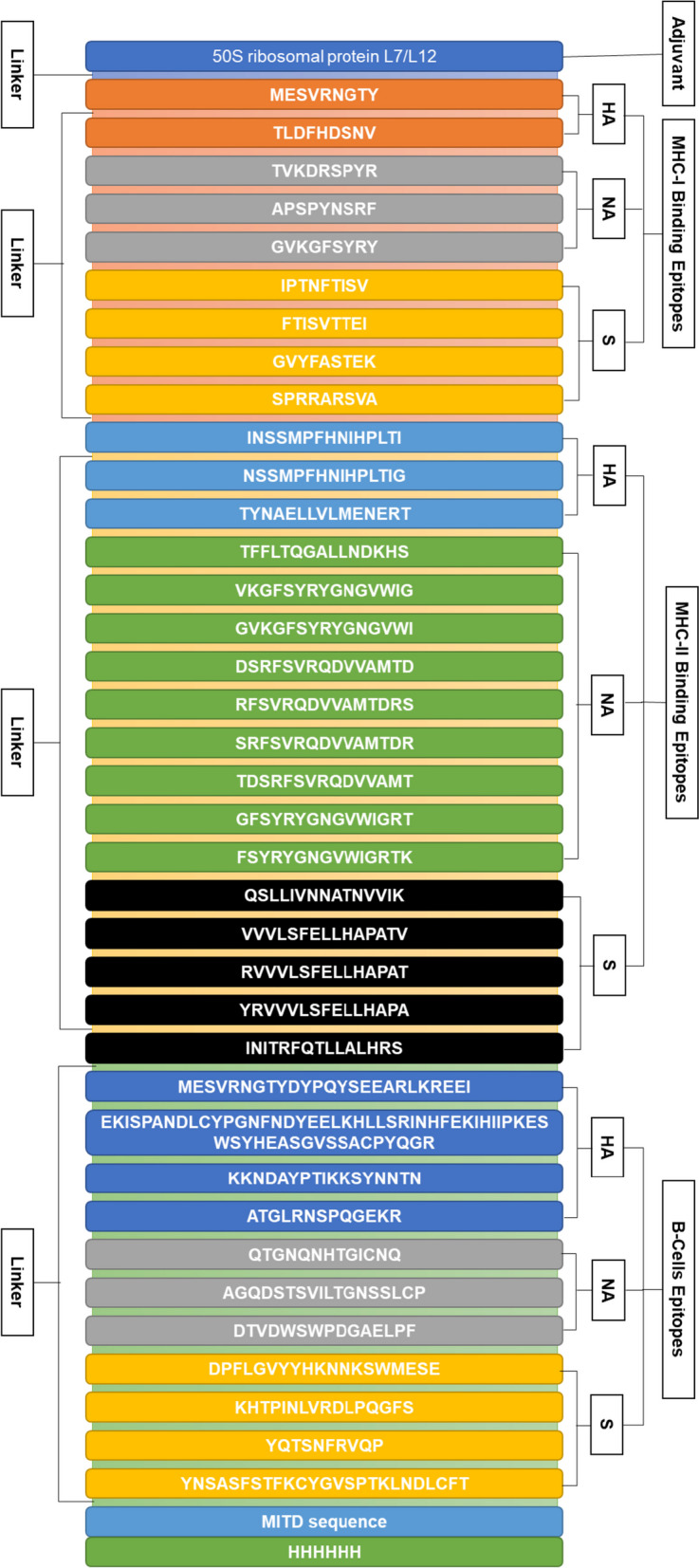
Combined vaccine construct of IAV and SARS-CoV-2 viruses with linkers and epitopes’ location

The constructed vaccine started from the N-terminal with 50S ribosomal protein adjuvant that linked with B-cell epitopes through the EAAAK linker. Four HA, three NA, and four S proteins of B-cell epitopes, two HA, three NA, and four S proteins of MHC-I epitopes, and three HA, nine NA, and five S proteins of MHC-II epitopes were elect for vaccine construction. At the C-end terminal of the vaccine, MITD sequence and 6x His tag were included in the vaccine construct (shown in Fig. 1).

### Physiochemical parameters, antigenicity, allergenicity, toxicity and solubility of vaccine construct

ProtParam online server evaluated the physiochemical properties of constructed vaccine (Table 6). VaxiJen 2.0 server confirmed the antigenicity of the constructed vaccine with a 0.6466 antigenicity score (Table 6). Moreover, AllerTop 2.0 and Toxinpred2 confirmed the non-allergic and non-toxic vaccine construct (Table 6). The solubility score of the constructed vaccine is higher than 0.5 (0.902), which confirmed the soluble expression of the vaccine in *E. coli* (Table 6).

**Table 6:** Physiochemical properties, antigenicity, allergenicity, toxicity and solubility of vaccine construct

Sr. No.	Physiochemical properties	Vaccine construct
**1**	Molecular weight	104,750.67
**2**	Amino acids number	975
**3**	Theoretical pI	9.76
**4**	Formula	C_4672_H_7284_N_1334_O_1369_S_22_
**5**	Extinction coefficients (M^−1^ cm^−1^, at 280nm)	116465
**6**	GRAVY	−0.400
**7**	II	27.53
**8**	AI	68.97
**9**	R^+^	119
**10**	R^-^	72
**11**	Estimated half-life	30 hours (mammalian reticulocytes, in vitro) >20 hours (yeast, in vivo). >10 hours (*E. coli*, in vivo)
**12**	Antigenicity Score	0.6466
**13**	Antigenicity	Antigen
**14**	Allergenicity	Non-allergen
**15**	Toxicity	Non-toxin
**16**	Solubility score	0.902

### Secondary and tertiary structures modeling

**Fig. 2:**
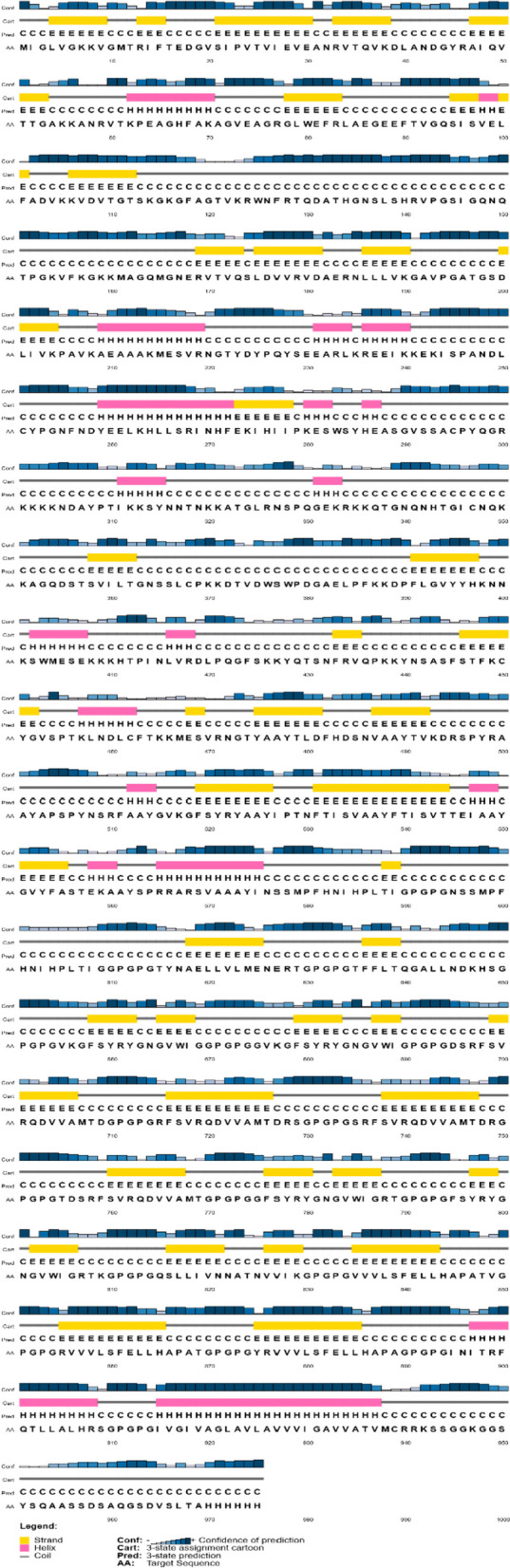
The secondary structure of the combined vaccine construct of IAV and SARS-CoV-2 viruses shown in the PSIPRED Cartoon

**Fig. 3:**
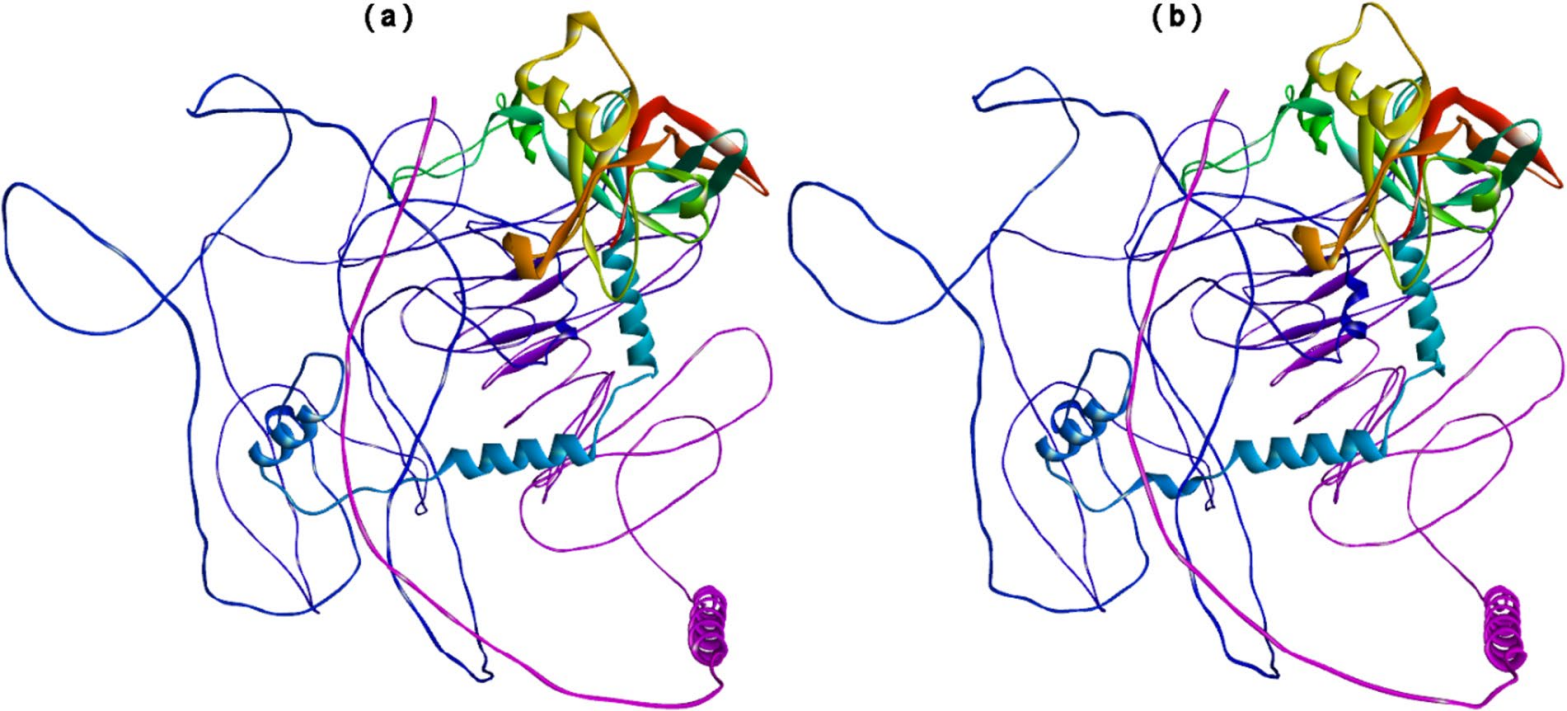
The 3D structure of conjugate vaccine construct of IAV and SARS-CoV-2 viruses predicted by ColabFold that uses AlphaFold2 and Alphafold2-multimer (**a**) and then refined by GalaxyRefine (**b**)

The SOPMA web server calculated different parameters necessary for the secondary structure of the constructed vaccine. Table 7 indicates the presence of alpha helix, extended strand, beta-turn, and random coil in the constructed vaccine. Although the percentage of the random coil is higher (45.64%) than the other predicted secondary structure of constructed vaccine for IAV and SARS-CoV-2 viruses, in parallel, PSIPRED results (Fig. 2) represent the location and confidence of prediction of the helix, extended strands, beta-turn, and random coil in the constructed conjugate vaccine. Furthermore, ColabFold constructed five 3D models of the vaccine protein based on their C-score that range from −5 to 2 with bad to good quality. Figure 3(a) displays the tertiary structure of the conjugate vaccine protein of IAV and SARS-CoV-2 viruses.

**Table 7:** Estimation of various secondary structure parameters of combined vaccine construct of IAV and SARS-CoV-2 viruses

Sr. No.	Secondary structure parameters	Percentages
**1**	Alpha helix (%)	14.87
**2**	Beta bridge (%)	0
**3**	Pi helix (%)	0
**4**	3_10_ helix (%)	0
**5**	Extended strand (%)	29.85
**6**	Beta turn (%)	9.64
**7**	Ambiguous states (%)	0
**8**	Random coil (%)	45.64
**9**	Bend region (%)	0
**10**	Other states (%)	0

### Refinement and verification of 3D vaccine

**Fig. 4:**
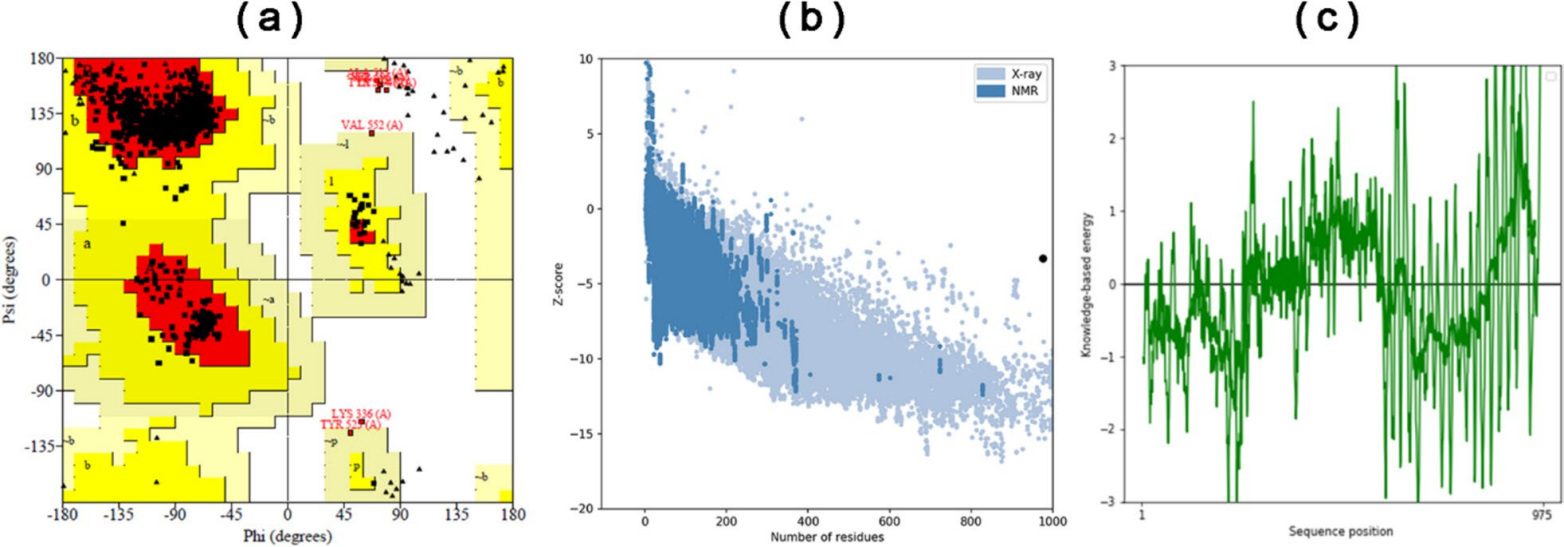
Validation of 3D structure of vaccine construct of IAV and SARS-COV-2 viruses. **a** Ramachandran plot validates the 3D predicted structure of the combined vaccine construct of IAV and SARS-COV-2 viruses. **b** The validation of the vaccine’s 3D structure was performed using ProSA-web. The refined model was assigned a *Z*-score of −3.33, indicating that it falls within the expected score range. **c** ProSA-web generates residue scores to evaluate the local quality of the model, and negative scores indicate the absence of any incorrect regions in the model’s structure

The selected 3D structure of the conjugate vaccine of IAV and SARS-CoV-2 viruses was refined by relaxing the structure with the procedure of MDS using GalaxyRefine online web server (Fig. 3b). The 3D structure of the conjugate vaccine was then validated with a Ramachandran plot and *Z*-scores. Figure 4(a) represents the Ramachandran plot of the conjugate vaccine. The R-factor of the Ramachandran plot with more than 90% of the residues in the most favored region is considered a good model. In the case of the 3D model of conjugate vaccine, residues of more than 90% (94.7%) were present in the most favored region of the Ramachandran plot.

Furthermore, the Ramachandran plot furnished details concerning residues located in the additionally allowed area (4.5%), permissibly allowed region (0.3%), the overall count of non-proline and non-glycine residues (786), the count of terminal residues (excluding proline and glycine) (2), the count of glycine residues (120), and the count of proline residues (67). Meanwhile, residues in the disallowed region of the Ramachandran plot were only 0.6%. Additionally, the *Z*-score of the conjugated vaccine (−3.33) was computed using the ProSA tool (Fig. 4b). Meanwhile, Fig. 4c shows the local model quality of each sequence position with the knowledge-based energy of the 3D model conjugated vaccine.

### Discontinuous B-cell epitope prediction of vaccine construct

**Fig. 5:**
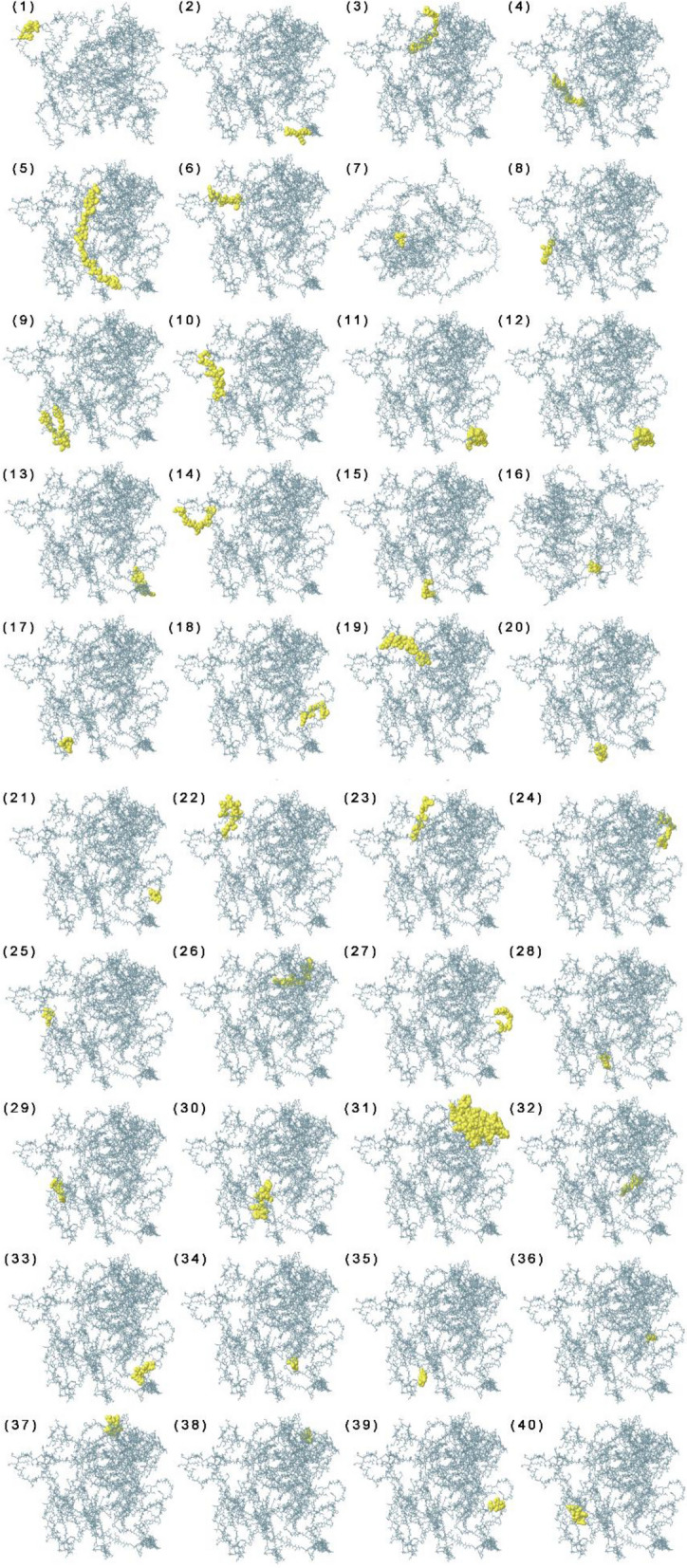
Discontinuous B-cell epitopes mapping on the vaccine construct (1–40) yellow area of the vaccine showing each discontinuous B-cell epitope containing residues from 3 to 78 with score values from 0.517 to 0.987. The number and types of residues and scores of each discontinuous B-cell epitope are mentioned in Table S4 from supplementary material

The presence of significant B-cell epitopes is essential in the vaccine to activate humoral immunity for the secretion of antibodies and cytokines against the foreign antigen. ElliPro tool confirmed the presence of 16 linear B-cell epitopes in the vaccine with 5–115 amino acid residues whose scores ranged from 0.508 to 0.833. In parallel, 40 discontinuous B-cell epitopes with residues ranging from 3 to 78 were confirmed in the vaccine, with score values from 0.517 to 0.987, as shown in Fig. 5. Tables S3 and S4 from supplementary material show the numbers, types of residues, and scores of each linear and discontinuous B-cell epitope, respectively.

### Molecular docking and molecular simulation

**Fig. 6:**
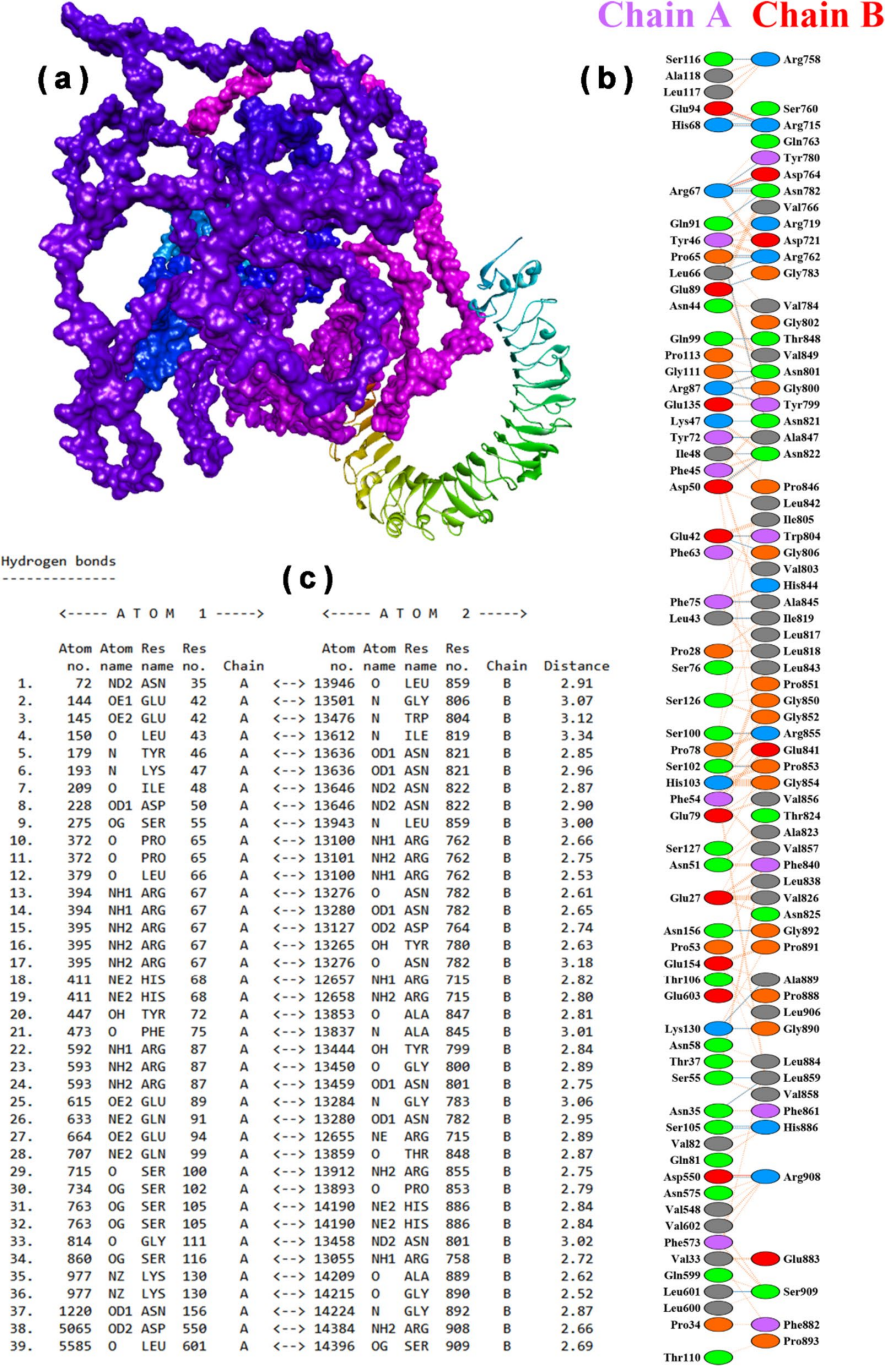
Best vaccine-TLR3 docking result obtained from ClusPro 2.0. **a** Docking position of vaccine with TLR3 (complex with cartoon solvent ribbon size surface is the vaccine, and ribbon structure is receptor). **b** Interactions between the residues of vaccine with TLR3. **c** Atoms and functional groups of vaccine residues interacting with atoms and functional groups of TLR3 residues

The vaccine-TLR3 docking was accomplished by ClusPro 2.0, which estimated the binding affinity of 30 different complexes. The best vaccine-TLR3 dock complex with binding affinity −1513.9 kcal/mol was visualized in PyMol and then Discovery Studio having 39 hydrogen bonds and 514 non-bonded contacts (Table 8), as shown in Fig. 6.

**Table 8:** The interface residues number, hydrogen bonds, salt bridges, and non-bonded contact with interface area, binding affinity, hydrophobic-favored binding affinity, and van-der Waal and electrostatic binding of best vaccine-TLR3 docking complex obtained from ClusPro 2.0

Docking	No. of interface residues	Interface area (Å2)	Binding affinity (kcal/mol)	Hydrophobic-favored binding affinity (kcal/mol)	Van-der Waal and electrostatic binding affinity (kcal/mol)	No. of hydrogen bonds	No. of salt bridges	No. of non-bonded contacts
TLR4	62	2986	−1513.9	−1842	−306.8	39	3	514
Vaccine	65	3105

**Fig. 7:**
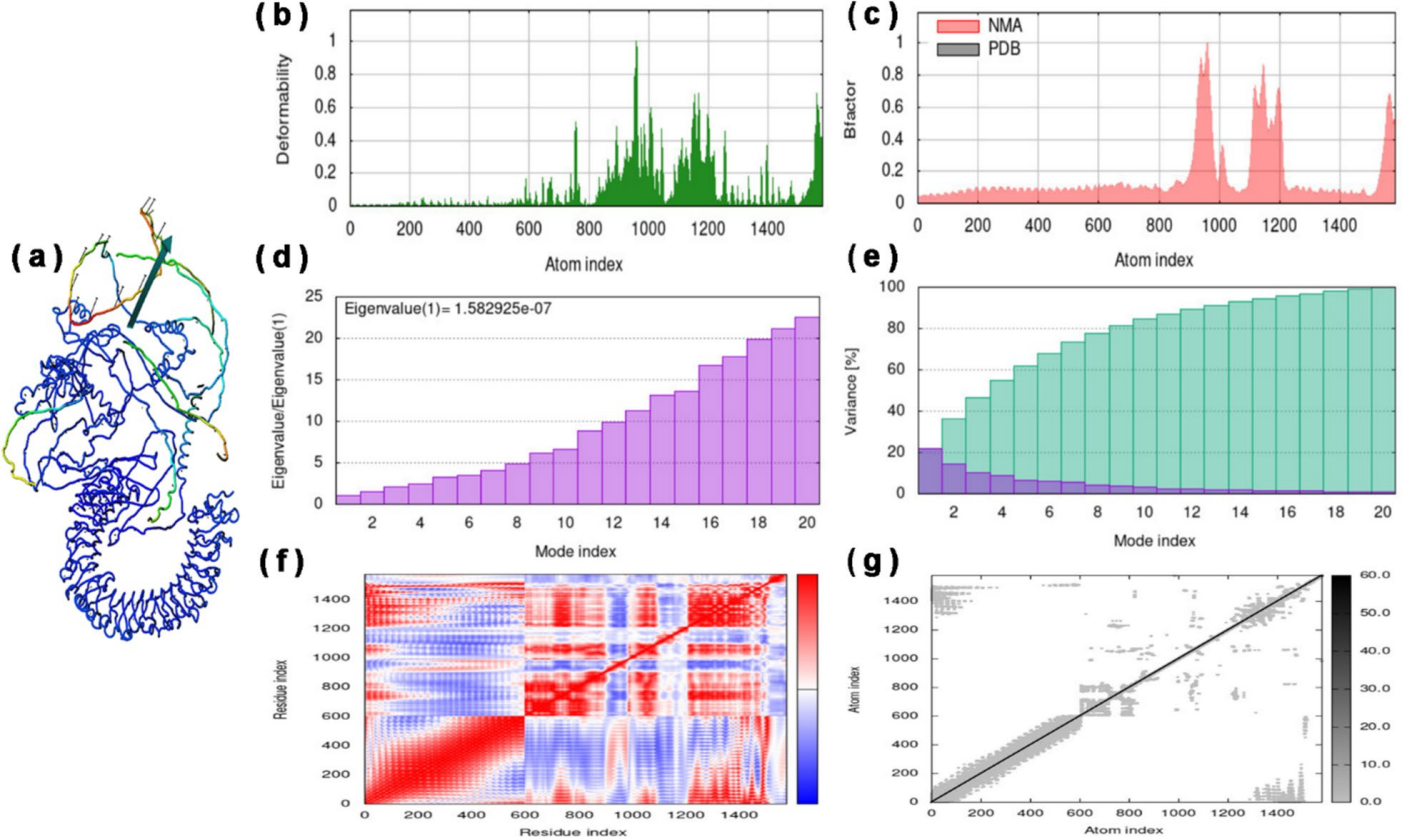
MDS results of best vaccine-TLR3 docked complex obtained by using iMODs server. **a** NMA mobility of vaccine-TLR3 complex showing with arrows. **b** Deformability of vaccine-TLR3 complex compared with atom index. **c** B-factor showing only NMA result with atom index. **d** Eigenvalues (1) = 1.582925e-07 compared with mode index. **e** Percentage variance (violet color for individual variances and cyan color for cumulative variances) of vaccine-TLR3 complex compared with mode index. **f** Covariance map of the vaccine-TLR3 complex for residue index (blue for anti-correlated motions, red for correlated motions, and white for uncorrelated motions). **g** Elastic network map for atomic index (darker gray regions for stiffer regions)

Furthermore, based on dynamics and normal modes, the mobility and stability of the vaccine-TLR3 docked complex was analyzed by the iMODS tool. The NMA mobility of residues and full vaccine-TLR3 complex was shown with small and large arrows, respectively (Fig. 7a). Similarly, the atomic index deformability of the vaccine-TLR3 complex is represented in Fig. 7b. The flexibility of a protein’s backbone is primarily influenced by the probability of a particular residue being distorted, and residues with higher flexibility scores are likely to be located at chain hinges, as depicted in the chain hinge diagram (Fig. 7b). The docked complex mobility is associated with the B-factor values obtained from NMA, which correlates with the RMS (root mean square) value (Fig. 7c). The eigenvalue of the vaccine-TLR3 docked complex, calculated as 1.582925e-07 (as shown in Fig. 7d), represents the overall rigidity of the complex. A lower eigenvalue implies that the protein complex is more easily deformed. The graph of variance exhibits the proportional contribution of each normal mode’s variance to the equilibrium movements, where collective and individual variances are represented by cyan and violet bars, respectively (shown in Fig. 7e). The patterns of the mobility of a particular molecule region are shown in the covariance graph, where white, blue, and red colors correspond to anti-related, uncorrelated, and related movements, respectively (as depicted in Fig. 7f). The elastic network graph displays the springs that link pairs of atoms, with each dot representing one spring. The stiffness of the springs is indicated by the color of the dot, with darker gray dots representing stiffer springs (Fig. 7g).

### Optimization of codons and in silico cloning

**Fig. 8:**
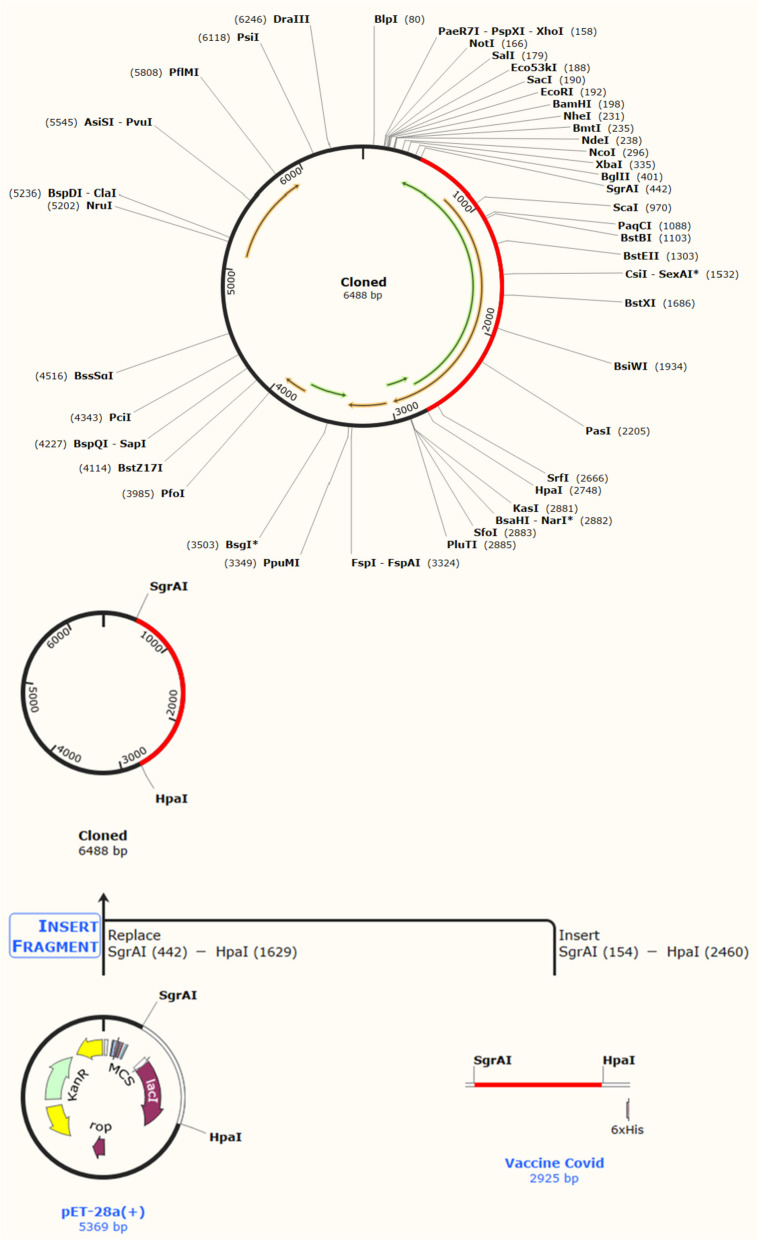
Insertion of improved and optimized vaccine into *E. coli* expression vector pET-28a (+) for in silico cloning using SnapGene 4.2 software. The red color represents the gene of interest between SgrAI (442) and HpaI (2460), and the remaining black color shows the expression vector pET-28a (+)

JCat tool customized the vaccine sequence for the *E. coli* K12 strain with 51.79% GC content, confirming the competent vaccine expression in *E. coli* bacteria with a 0.901 CAI (codon adaptation index) value. Moreover, the vaccine’s improved and optimized DNA sequence was infused in the *E. coli* vector PET28a(+) between the SgrAI and HpaI restriction sites, as represented in Fig. 8. The clone with a length of 6.4 kbp was constructed. Following vaccine expression, the recombinant vaccine was purified through affinity chromatography by incorporating a 6-histidine tag at both termini.

### mRNA vaccine prediction for the secondary structure

**Fig. 9:**
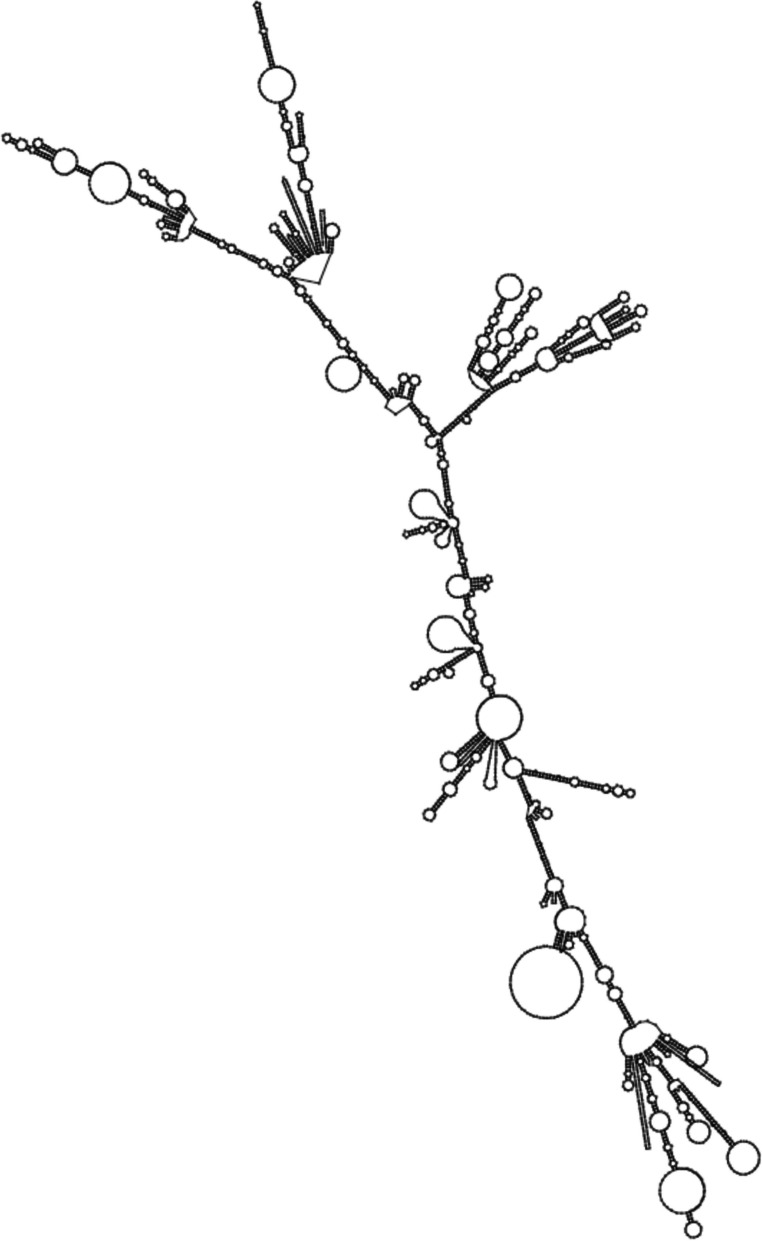
Predicted centroid secondary structure of mRNA of the vaccine construct

The optimized DNA sequence of the vaccine construct was then converted into an RNA sequence for the mRNA vaccine construct. The secondary structure of the vaccine mRNA construct was generated using RNAfold, with a minimum free energy of −939.90 kcal/mol, as depicted in Fig. 9.

### Immune simulation analysis

**Fig. 10:**
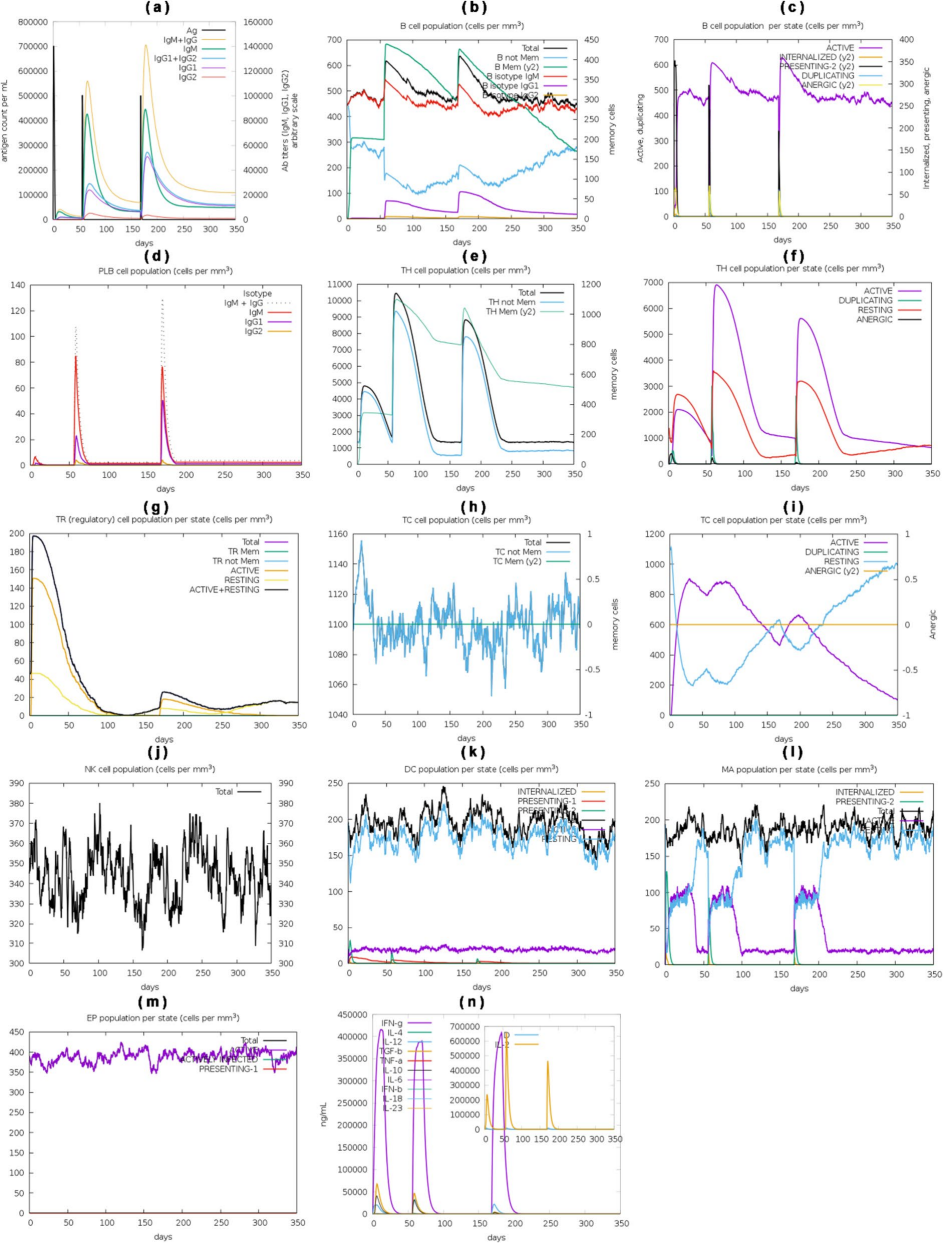
The designed vaccine was subjected to an in silico immune response simulation as an antigen with 3 shots for 350 days. The simulation involved evaluating several parameters, including the (**a**) antigen and antibody levels, (**b**) population of B-cells, (**c**) B-cell population density (cells per mm^3^), (**d**) plasma B-cell population density (cells per mm^3^), (**e**) helper T-cell population density (cells per mm^3^), (**f**) helper T-cell population density per state (cells per mm^3^), (**g**) Th1 cell population, regulatory T-cell population density per state (cells per mm^3^), (**h**) cytotoxic T-cell population density (cells per mm^3^), (**i**) cytotoxic T-cell population density per state (cells per mm^3^), (**j**) natural killer cell population density (cells per mm^3^), (**k**) dendritic cell population density per state (cells per mm^3^), (**l**) macrophage population density per state (cells per mm^3^), (**m**) epithelial presenting cells population density per state (cells per mm^3^), and (**n**) concentration of interleukins and cytokines. The simulation results were assessed using the Simpson index (D) to evaluate the immune response

C-ImmSim confirmed the significant stimulation of primary immune response with the gradual increase in the level of immunoglobulins such as IgG, IgG1, IgG2, and IgM after the first, second, and third doses. However, the concentration of immunoglobulins was higher just after the vaccine’s inoculation, which decreased with time, although immunoglobulins concentration was significantly higher after the third dose. In contrast, antigen concentration reduced during and after the second and third doses of the vaccine (as shown in Fig. 10a). Also, the active and total B-cell population was sustainably elevated, as shown in Fig. 10b, c, while plasma B-cells concentration increased for some days after the vaccination (Fig. 10d). Similarly, the active total helper T-cells were elevated and sustained after the inoculation (Fig. 10e, f). The active and resting helper regulatory T-cells concentration was high after the vaccine’s first shot, progressively reducing over time (Fig. 10g).

Meanwhile, the cytotoxic helper T-cells concentration varies with time (Fig. 10h), and their active form decreases with constant energy after vaccination doses (Fig. 10i). Respectively, the population of natural killer cells also fluctuated during the vaccination process (Fig. 10j). The concentrations of dendritic cells, macrophages, and epithelial presenting cells were evaluated in cells per mm^3^, shown in Fig. 10k–m. The activation of different cells, different cytokines, and interleukin concentrations were also elevated after the vaccine (Fig. 10n).

## Discussion

With the confirmation of flurona in various countries like Israel, the USA, and Brazil, discussion about the co-administration of vaccines has significantly started. This debate has been further promoted with results of optimized vaccine coverage, reduced vaccination-related visits, limited potential adverse effects, and recommendations for after-clinical testing [93]. In this respect, it is crucial to protect vulnerable individuals against both IAV and SARS-CoV-2 infections simultaneously to ease the burden on healthcare services and prevent severe disease caused by co-infection [94]. In response to this need, the WHO recommended the co-administration of both vaccines in October 2021 [95] based on preliminary clinical evidence suggesting acceptable safety and immunogenicity [96, 97]. While many countries are now promoting the co-administration of SARS-CoV-2 and influenza vaccines, further research is needed to understand their safety, immunogenicity, and effectiveness. Such research would help instill confidence in vaccinators among the general public, leading to increased vaccine coverage and better overall protection [93].

The co-administration of two vaccines elicited an idea to make a multi-epitope-containing vaccine with the antigenic, non-toxic, and non-allergic properties of different viruses, such as IAV and SARS-CoV-2. For designing a vaccine against the IAV, NA, and HA, proteins are the surface glycoproteins that play a significant role in attachment and replication [98, 99]. HA protein also triggers the host immune system to produce antibodies that neutralize the virus. Therefore, most IAV vaccines target the HA protein as a whole or subunit. Meanwhile, the presence of NA in the IAV vaccine stimulates producing antibodies that can block the activity of the NA protein, thus preventing the release and spread of the virus. Therefore, including NA in the IAV vaccine is crucial for its efficacy in protecting against influenza infection [100–103].

Similarly, the S protein plays a crucial role in developing SARS-CoV-2 vaccines, the key antigen of SARS-CoV-2 and responsible for binding to the host cell receptor, facilitating viral entry. Therefore, by targeting the S protein, vaccines aim to elicit an immune response that can prevent the virus from infecting human cells [104–107]. Thus, the present study aims to construct a non-toxic and non-allergic protein and mRNA-based conjugate vaccine containing multiple epitopes of two proteins, i.e., NA and HA of IAV and an S protein of SARS-CoV-2, using artificial intelligence. Furthermore, the constructed conjugate vaccine was evaluated with Bioinformatic tools for its interaction with immunity-triggering receptors and real-life immunogenic profiling.

The sequences of the Israeli strain of NA and HA of IAV and S protein of SARS-CoV-2 were multiply aligned to identify their conserved regions. Then, their antigenicity was confirmed for designing a conjugate vaccine against two viruses in *Homo sapiens*. As all the HA, NA [108, 109], and S protein [110] are transmembrane, most of the sequences were identified to be present outside the lipid membranes of both viruses (as shown in Table 1); a similar process of selecting sequences has been reported in other studies [81, 111–113]. In consideration of the ongoing mutational dynamics inherent in RNA viruses, namely IAV, and SARS-CoV-2, leading to the emergence of distinct serotypes, we employed a computational approach focusing on the identification of conserved regions within the NA and HA sequences of IAV and the S protein sequence of SARS-CoV-2. These conserved regions were utilized to predict antigenic T- and B-cell epitopes. Furthermore, the physiochemical properties of each selected HA, NA, and S protein were also determined (Table 2).

The MHC-1 binding epitopes were predicted with the NetMHCpan EL4.1 method. Two MHC-1 binding epitopes (MESVRNGTY and TLDFHDSNV) in HA protein and three MHC-1 binding epitopes (TVKDRSPYR, APSPYNSRF, and GVKGFSYRY) in NA protein of IAV with antigenic, non-allergic, and non-toxic properties having Israeli population coverage of 89.39% were found and selected for vaccine designing (as shown in Table 3). Similarly, four antigenic, non-toxic, and non-allergic MHC-1 binding epitopes (IPTNFTISV, FTISVTTEI, GVYFASTEK, and SPRRAPSVA) were selected from the sequence of S protein of SARS-CoV-2 for designing vaccine (Table 3).

Accordingly, MHC-II binding epitopes were predicted with IEDB recommended 2.22 method. Three MHC-II binding epitopes (INSSMPFHNIHPLTI, NSSMPFHNIHPLTIG, and TYNAELLVLMENERT) of HA protein and nine MHC-II epitopes (TFFLTQGALLNDKHS, VKGFSYRYGNGVWIG, GVKGFSYRYGNGVWI, DSRFSVRQDVVAMTD, RFSVRQDVVAMTDRS, SRFSVRQDVVAMTDR, TDSRFSVRQDVVAMT, GFSYRYGNGVWIGRT, and FSYRYGNGVWIGRTK) of NA protein of IAV were found antigenic, non-allergic, and non-toxic with 68.79% of Israeli population coverage for vaccine designing (Table 4). Likewise, in the S protein of SARS-CoV-2, five antigenic, non-toxic, and non-allergic MHC-II epitopes such as QSLLIVNNATNVVIK, VVVLSFELLHAPATV, RVVVLSFELLHAPAT, YRVVVLSFELLHAPA, and INITRFQTLLALHRS were selected for the vaccine designing as mentioned in Table 4.

The MHC-I and MHC-II epitopes were relevant for the T-cells. Meanwhile, the BepiPred Linear Epitopes Prediction 2.0 method was used to predict B-cell epitopes. In this respect, four B-cell epitopes of HA, three epitopes of NA, and four epitopes of S protein were found suitable for the conjugate vaccine designed against IAV and SARS-CoV-2 (Table 5).

Following reported studies [81, 82, 92, 114–116], a vaccine consisting of 975 residues with a molecular weight of 104.75 kilodaltons (kDa), a basic pI of 9.76, an antigenic score of 0.6466, non-toxic and non-allergic properties, and solubility of 0.902 (Table 6) was developed by incorporating selected MHC-I, MHC-II, and B-cell epitopes, linkers, adjuvants, MITD sequence, and 6x His Tag. Then, the secondary (Table 7 and Fig. 2) and tertiary structures (Fig. 3) of a protein-based conjugate vaccine against IAV and SARS-CoV-2 were determined. The 3D structure of the conjugate vaccine was also confirmed by the Ramachandran plot and *Z*-score (Fig. 4). Sixteen linear and forty discontinuous B-cells epitopes (Tables S3 and S4 from supplementary material and Fig. 5) confirmed the ability of the conjugate vaccine to activate the B-cells for antibody production.

After the inoculation of the vaccine, its primary goal is to activate the immune response against the foreign antigen. For this purpose, antigen (conjugate vaccine) first binds with the TLR3 receptor on the surface of different immune cells, such as dendritic cells, macrophages, natural killer cells, non-immune fibroblasts, and epithelial cells. TLR3 recognizes the foreign invaders and triggers a signaling cascade mechanism for activating innate and adaptive immune response and producing interferons and other pro-inflammatory cytokines. The molecular docking results of vaccine-TLR3 confirmed a significant −1513.9 kcal/mol of binding energy with 39 hydrogen bonds and 514 non-bonded contacts (Table 8 and Fig. 6). Our vaccine construct demonstrated notably enhanced binding affinity toward TLR3 in comparison to findings from previous investigations. For instance, in a study [117], a vaccine targeting SARS-CoV-2 exhibited a binding energy of −941.7 kcal/mol with TLR3. Similarly, another study [118] reported a binding affinity of −1324.9 kcal/mol between the vaccine and TLR3 in the context of SARS-CoV-2. Furthermore, a separate study [119] revealed a binding score of −1089 at the center, and the lowest energy observed in the TLR3-vaccine complex was −1258.7 kcal/mol against SARS-CoV-2. In parallel, a recent study reported a binding score of −1170.9 at the center, with the lowest energy observed in the TLR3-vaccine complex against IAV being −1403.8 kcal/mol [120]. Furthermore, the molecular simulation results showed the mobility, deformability, B-factor, 1.582925e-07 eigenvalues, variance, and covariance of the vaccine-TLR3 complex (Fig. 7).

The expression of the vaccine construct was then analyzed by in silico cloning, as shown in Fig. 8. In this study, the vaccine’s protein construct was converted into a DNA construct. The resulting conjugate vaccine DNA construct was 2925 base pairs (bp) in length, translating to an estimated molecular weight of approximately 100 kDa for the recombinant protein. However, due to its large size, the expression of this recombinant protein in the E. coli system posed challenges. Proper folding, assembly, post-translational modifications, and purification of the large protein were difficult to achieve in this system. Two main alternatives were considered to address these challenges. The first involved modifying the DNA construct to produce a truncated version of the protein. However, this approach risked losing the protein’s desired functionality and biological activity. The second alternative explored was the use of mRNA vaccines. In this approach, the mRNA encoding the target protein was synthesized and introduced into host cells, allowing the cells’ machinery to produce the protein. In order to facilitate this aim, the finalized DNA sequence was transcribed into mRNA, and its secondary structure was predicted, as depicted in Fig. 9. The use of mRNA vaccines offers a promising strategy to overcome the limitations associated with the expression of large recombinant proteins in the E. coli system. This in silico approach allows for the prediction of the mRNA’s secondary structure, aiding in developing a potential mRNA-based vaccine for the target protein. Further studies and characterization will be necessary to assess the mRNA-based vaccine’s functionality and immunogenicity and optimize its design for future applications.

Finally, the in silico immune response of the conjugate vaccine was validated by inoculating three vaccine shots of 1000 antigens with eight and then 24 weeks intervals after the 1st shot for a total of 350 days. The production of all the required immune cells, interferons, and other pro-inflammatory cytokines against the vaccine was produced with different concentrations at different times after the inoculation of the vaccine, as shown in Fig. 10.

Currently, few efforts have been made to suggest universal vaccines against IAV and SARS-CoV-2 viruses, and the development of such vaccines has not been successful. The futile experiments stem from the two viruses’ subtle differences in pathogenesis, host-pathogen interactions, and immune responses. Hence, the study has utilized a systemic immunoinformatics approach in this research to develop a potent multi-epitope-based IAV and SARS-CoV-2 conjugate vaccine. However, despite the potential of the immunoinformatics approach, there may be limitations due to the absence of a standard benchmark for vaccine development against IAV and SARS-CoV-2 and limited knowledge of their pathogenesis and adaptive immune systems response. Consequently, to evaluate the immunogenicity, efficacy, and safety of the newly developed vaccine, experimental validation is required both in vivo and in vitro.

## Conclusion

To identify a potential candidate for clinical trials, in silico vaccine design utilizing computational approaches was performed to construct an effective vaccine against NA and HA of IAV and S protein of SARS-CoV-2, to achieve good population coverage and immune response. By employing immuno-informatics techniques, T- and B-cell epitopes were predicted, while molecular docking was conducted with ClusPro, demonstrating a binding energy of −1513.9 kcal/mol, and the Ramachandran plot indicating a favored region of 94.7%. The vaccine construct was found to have good protein expression as determined by the SnapGene tool. Moreover, in silico trials demonstrated a strong immune response to the conjugate vaccine. The proposed vaccine construct fulfilled the criteria for antigenicity, allergenicity, toxicity, and other physicochemical properties, suggesting it is safe, although preclinical studies and authentication are required before experimental clinical trials can be conducted to confirm the study results.

### Supplementary Information

**Additional file 1.**

## Data Availability

The corresponding author (AE) can be contacted to obtained the available data which support the findings of this study.

## Abbreviations

IAV: Influenza A virus
WHO: World Health Organization
NA: Neuraminidase
HA: Hemagglutinin
S: Spike
COBALT: Constraint-based Multiple Alignment Tool
TMHMM 2.0: Transmembrane helices hidden Markov models
pI: Theoretical isoelectric point
AI: Aliphatic index
Ec: Extinction coefficients
GRAVY: Grand average of hydropathicity
II: Instability index
R-: Negatively charged residues
R+: Positively charged residues
IEDB: Immune Epitope Database
PSIPRED: PSI-blast-based secondary structure PREDiction
MDS: Molecular dynamics simulation
TLR3: Toll-like receptor 3
PDB: Protein Data Bank
MITD: HC I-targeting domain
NMA: Normal mode analyses
JCAT: Java Codon Adaptation Tool
PSSM: Position-specific scoring matrix
